# Cyr61/CCN1 Is Regulated by Wnt/β-Catenin Signaling and Plays an Important Role in the Progression of Hepatocellular Carcinoma

**DOI:** 10.1371/journal.pone.0035754

**Published:** 2012-04-23

**Authors:** Zhi-Qiang Li, Wei Ding, Shi-Jun Sun, Jun Li, Jing Pan, Chen Zhao, Wei-Ru Wu, Wei-Ke Si

**Affiliations:** 1 Department of Clinical Hematology, Third Military Medical University, Chongqing, China; 2 Department of Clinical Laboratory, KunMing General Hospital of PLA, KunMing, China; 3 Southwest Hospital, Third Military Medical University, Chongqing, China; Northwestern University Feinberg School of Medicine, United States of America

## Abstract

Abnormal activation of the canonical Wnt signaling pathway has been implicated in carcinogenesis. Transcription of Wnt target genes is regulated by nuclear β-catenin, whose over-expression is observed in Hepatocellular Carcinoma (HCC) tissue. Cyr61, a member of the CCN complex family of multifunctional proteins, is also found over-expressed in many types of tumor and plays dramatically different roles in tumorigenesis. In this study, we investigated the relationship between Cyr61 and β-catenin in HCC. We found that while Cyr61 protein was not expressed at a detectable level in the liver tissue of healthy individuals, its expression level was elevated in the HCC and HCC adjacent tissues and was markedly increased in cancer-adjacent hepatic cirrhosis tissue. Over-expression of Cyr61 was positively correlated with increased levels of β-catenin in human HCC samples. Activation of β-catenin signaling elevated the mRNA level of Cyr61 in HepG2 cells, while inhibition of β-catenin signaling reduced both mRNA and protein levels of Cyr61. We identified two TCF4-binding elements in the promoter region of human Cyr61 gene and demonstrated that β-catenin/TCF4 complex specifically bound to the Cyr61 promoter *in vivo* and directly regulated its promoter activity. Furthermore, we found that over-expression of Cyr61 in HepG2 cells promoted the progression of HCC xenografts in SCID mice. These findings indicate that Cyr61 is a direct target of β-catenin signaling in HCC and may play an important role in the progression of HCC.

## Introduction

Hepatocellular carcinoma (HCC) is the fifth most common malignancy and the third deadliest cancer worldwide. Nearly 78% of the global HCC were reported in Asian countries [1]. According to the WHO report, China has the highest incidence and mortality of HCC in the world. Since the 1990s, HCC has become the second leading cause of cancer death in China.

Multiple factors and signaling pathways have been shown to contribute to the tumorigenesis of HCC [2]. Wnt/β-catenin signaling pathway plays an essential role in all phases of liver development and maturation. The aberrant activation of canonical Wnt/β-catenin signaling was shown to contribute to the HCC development [3], [4], [5]. In the absence of Wnt, intracellular β-catenin is either bound to cadherins at cell adhesion junctions or rapidly degraded in the cytosol by a multi-protein complex consisting of β-catenin, glycogen synthase kinase-3β (GSK-3β), adenomatous polyposis coli (APC), and Axin. When Wnt binds to the cell surface receptor, GSK-3β is inactivated, thereby releases β-catenin, which translocates to the nucleus, binds to T-cell factor 4/lymphocyte enhancer factor (TCF4/LEF), and targets Wnt-responsive genes. Several known Wnt/β-catenin target genes were found being expressed in HCC, including NOTUM [6], GS [7] and TBX3 [8]. Moreover, 33 novel candidate target genes were discovered expressed in hepatoma cells and were considered to be involved in multiple biological processes such as signaling transduction, inflammation, cell growth, proliferation and protein biosynthesis [9].

Cysteine-rich protein 61 (Cyr61/CCN1), a member of the CCN growth factor family, is a secreted, integrin-binding protein that regulates multiple cellular activities such as cell adhesion, migration, proliferation, survival and apoptosis [10]. Alterations in expression of Cyr61 were seen in many human tumors; however, its role varies in different tumor types [11]. Cyr61 was shown in some studies to promote tumorigenesis, progression and invasion in cancers including gliomas [12], gastric cancer [13], breast cancer [14], and prostatic carcinoma [15]. On the other hand, it was found to play roles such as inducing apoptosis, inhibiting tumor growth in non-small-cell lung cancer [16], cervix cancer [17] and endometrial cancer [18]. Its role in HCC is also controversial. While some laboratories showed that Cyr61 was down-regulated in HCC and that Cyr61 suppressed cell proliferation in HCC cell lines [19], [20], others found that expression of Cyr61 was not significantly different in HCC tissue compared to surrounding non-tumor tissue [21]. There is also a study showing that Cyr61 is over-expressed in HCC tissue [22].

There is evidence that expression of some members of CCN family, namely CTGF/CCN2 and WISP-1/CCN4, are regulated by Wnt/β-catenin signaling pathway [23], [24]. Our previous studies have demonstrated that Cyr61 is regulated by canonical Wnt signaling in mesenchymal stem cells [25]. Other studies found that Cyr61 can activate the β-catenin-TCF4 signaling pathway in gliomas cells [26] and non-small-cell lung cancer [16]. So, the relationship between Cyr61 and Wnt/β-catenin signaling pathway is complex in different cells. However, it is not clearly illustrated in HCC cell lines. In this report, we study the relationship between Wnt/β-catenin signaling and Cyr61 expression in HCC. We found that Cyr61 was over-expressed in HCC and its expression levels were positively correlated with the activation of β-catenin protein. Furthermore, we demonstrated that Cyr61 is a direct target gene of β-catenin signaling in HepG2 cells and over-expression of Cyr61 promotes the proliferation of HepG2 cells both *in vitro* [27] and *in vivo*. Our finding strongly suggests that Cyr61 is regulated by β-catenin in HCC and has multiple functions in the genesis and the progression of HCC.

## Results

### Expression of Cyr61 protein in normal liver tissue, hepatic cirrhosis, HCC and tumor-adjacent tissue

We previously reported that elevated Cyr61 mRNA levels were found in the HCC tissue compared to those found in the normal tissue by RT-PCR analysis. Moreover, we observed that the expression of Cyr61 was closely associated with some clinical manifestations, including the number of cancer nodules, degree of tumor differentiation, and venous infiltration. However, the expression did not correlate with some other clinical observations, such as the size of tumor, metastasis, cancer embolus and AFP concentration [28].

**Figure 1:**
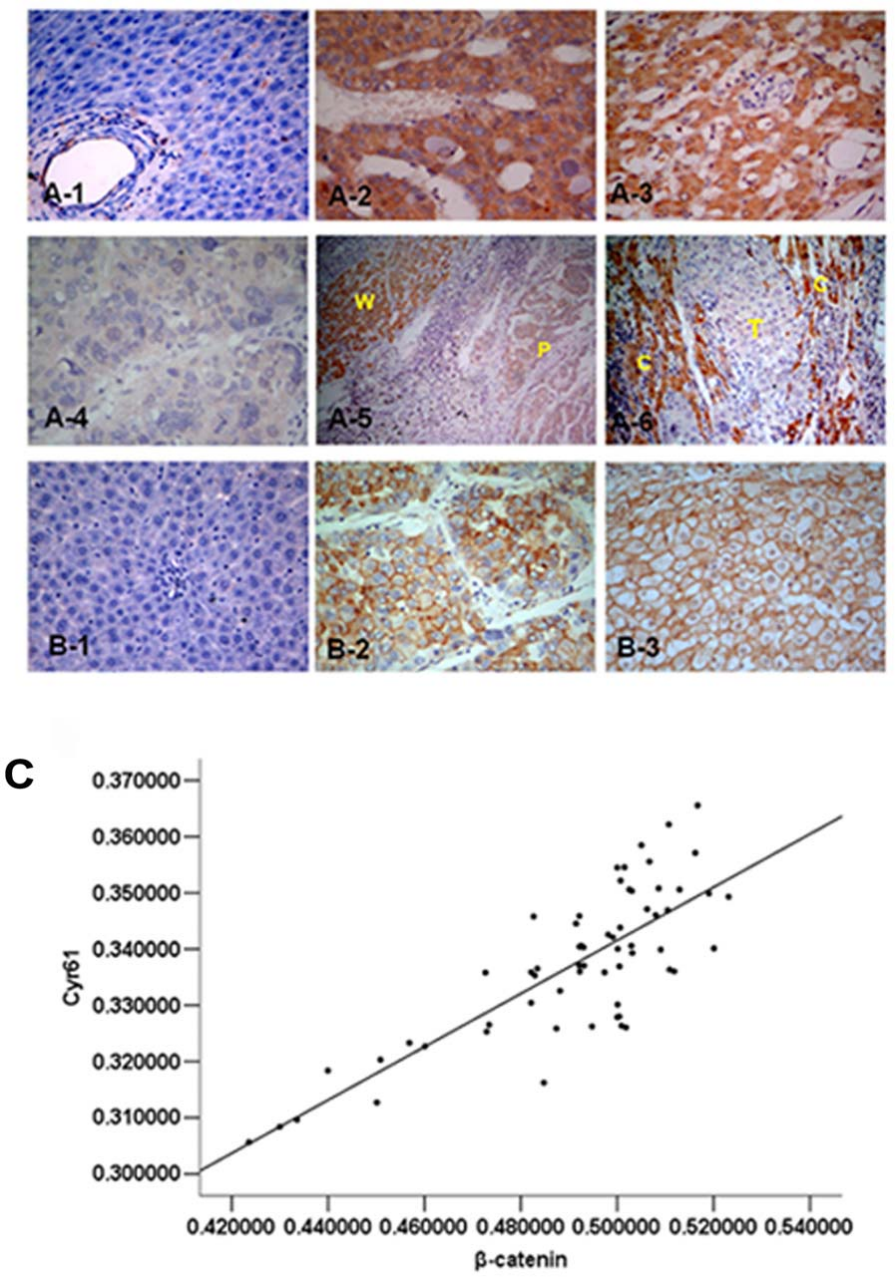
Cyr61 and β-catenin protein expression in tissue samples are detected by immunohistochemistry. (**A**), A-1: Expression of Cyr61 in normal liver tissues was negative (400×); Expression of Cyr61 in well-differentiated HCC (A-2) was higher than in poorly differentiated HCC (A-4) (400×); A-3: Expression of Cyr61 was positive in HCC adjacent tissue (400×); A-5: Expression of Cyr61 in well-differentiated tumor regions (W) was higher than poorly differentiated areas (P) at the same section (100×); A-6: Overexpression of Cyr61 in hepatic cirrhosis of adjacent cancer areas (C), and loss of expression in HCC (T) (200×); W, well-differentiated tumor; P, poorly differentiated tumor; C, hepatic cirrhosis; T, tumor. (**B**), B-1: Expression of β-catenin in normal liver tissues was negative; B-2: Expression of β-catenin in tumor tissue; B-3: Expression of β-catenin in HCC adjacent tissue. (**C**), Correlation between Cyr61 and β-catenin protein expression levels. *r = 0.793*, *P<0.01*.

There are reports by other laboratories indicating that expression of Cyr61 is down regulated or is not significantly different in HCC tissue compared with that in the normal tissue [19], [21]. We analyzed a greater number of samples with a different approach to look into the discrepancy. Immunohistochemistry staining was employed to study the expression of Cyr61 protein in normal liver tissue (n = 5), hepatic cirrhosis of cancer-adjacent tissue (n = 30), HCC (n = 62) and the HCC adjacent tissue (n = 27). We found that Cyr61 was expressed in 91.4% of HCC tissues and in 97.6% of adjacent tissue. However, it was not expressed or expressed at a very low level in normal liver tissue (Figure 1: A-1). Cyr61 expression was significantly higher in HCC adjacent tissue compared to tumor tissue (Figure 1: A-3; A-4, *P*<0.05), and was mainly located in cytoplasm (Figure 1: A). In tumors at different differentiation stages, the Cyr61 protein level was found significantly higher in well-differentiated HCC than in the poorly differentiated HCC (Figure 1: A-2; A-4, *P*<0.01). This phenomenon was also observed within the same tumor where stronger staining was found in more differentiated tumor cells (Figure 1: A-5). Furthermore, significant increases in Cyr61 expression levels were observed in the cirrhotic tissues adjacent to HCC compared to HCC itself (Figure 1: A-6, *P*<0.01).

### Cyr61 over-expression is tightly linked to activated β-catenin signaling in human HCC tissues

Expression of Cyr61 and β-catenin were negative or weak in normal liver tissue (Figure 1: A-1; B-1). Similar to the changes of expression level of Cyr61 in HCC and normal liver tissue, β-catenin expression level in HCC and HCC adjacent tissue was significantly increased in comparison to normal liver tissue (Figure 1: B). β-catenin was mainly localized at the cell membrane in HCC adjacent tissue (Figure 1: B-3), but it was also found localized in the cytoplasm and/or nucleus in tumor tissue (Figure 1: B-2). Cyr61 was localized in the cytoplasm of both tumor and adjacent tissues (Figure 1: A). Our findings point to a positive correlation between high Cyr61 expression level and over-expression of β-catenin protein (r = 0.793, *P*<0.01) (Figure 1C).

### Cyr61 expression levels are influenced by the alteration of β-catenin expression

**Figure 2:**
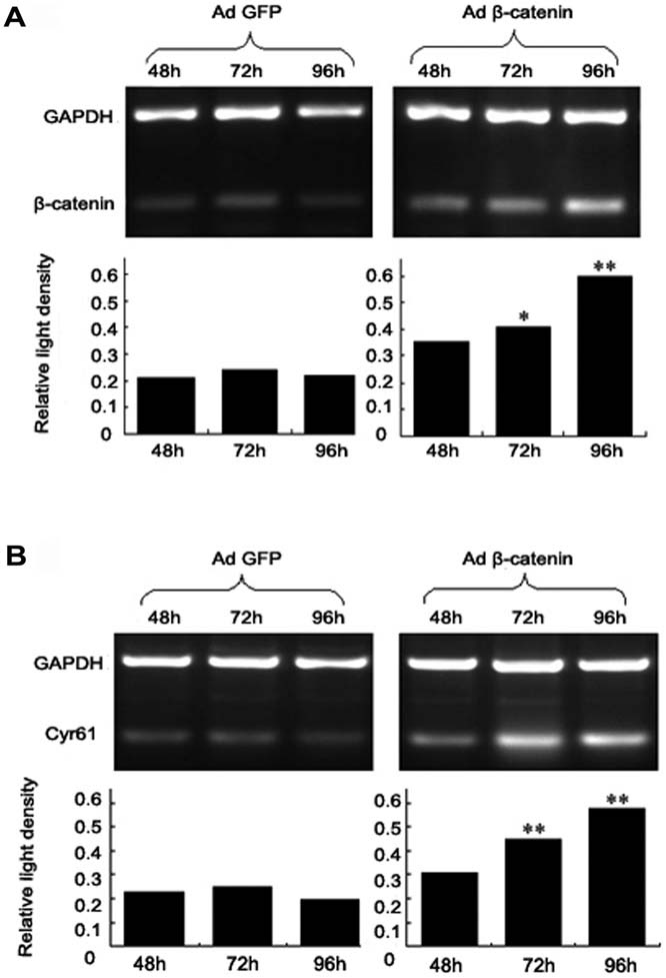
Over-expression of β-catenin up-regulates Cyr61 expression. HepG2 cells were infected with Adβ-catenin or AdGFP at 24 hrs after plating. Cells were harvested at 48, 72, and 96 hours after continuous incubation. RT-PCR was performed to detect the mRNA levels of β-catenin and Cyr61. PCR products for β-catenin and GAPDH (**A**) or Cyr61 and GAPDH (**B**) from AdGFP and Adβ-catenin infected cells at the indicated time points were resolved on agarose gel. Relative quantity of β-catenin or Cyr61 mRNA was calculated based on relative intensity (β-catenin OD/GAPDH OD or Cyr61 OD/GAPDH OD). *, *P<0.05*, **, *P<0.01*.

The correlation of expression between β-catenin and Cyr61 in HCC samples leads us to postulate that β-catenin may regulate Cyr61 expression. HCC cell line HepG2 was used to determine whether the expression of Cyr61 could be influenced by β-catenin. We infected HepG2 cells with adenovirus expressing β-catenin, and assessed the Cry61 mRNA levels at various time points post infection. An increased expression of β-catenin was observed at 48, 72 and 96 hrs after adenovirus Adβ-catenin infection, which was not seen in cells infected with a control virus, AdGFP (Figure 2A). Assessment of Cyr61 mRNA levels in the same samples revealed that the expression of endogenous Cyr61 also increased at a similar time course in the cells infected with Adβ-catenin but not the AdGFP virus (Figure 2B).

**Figure 3:**
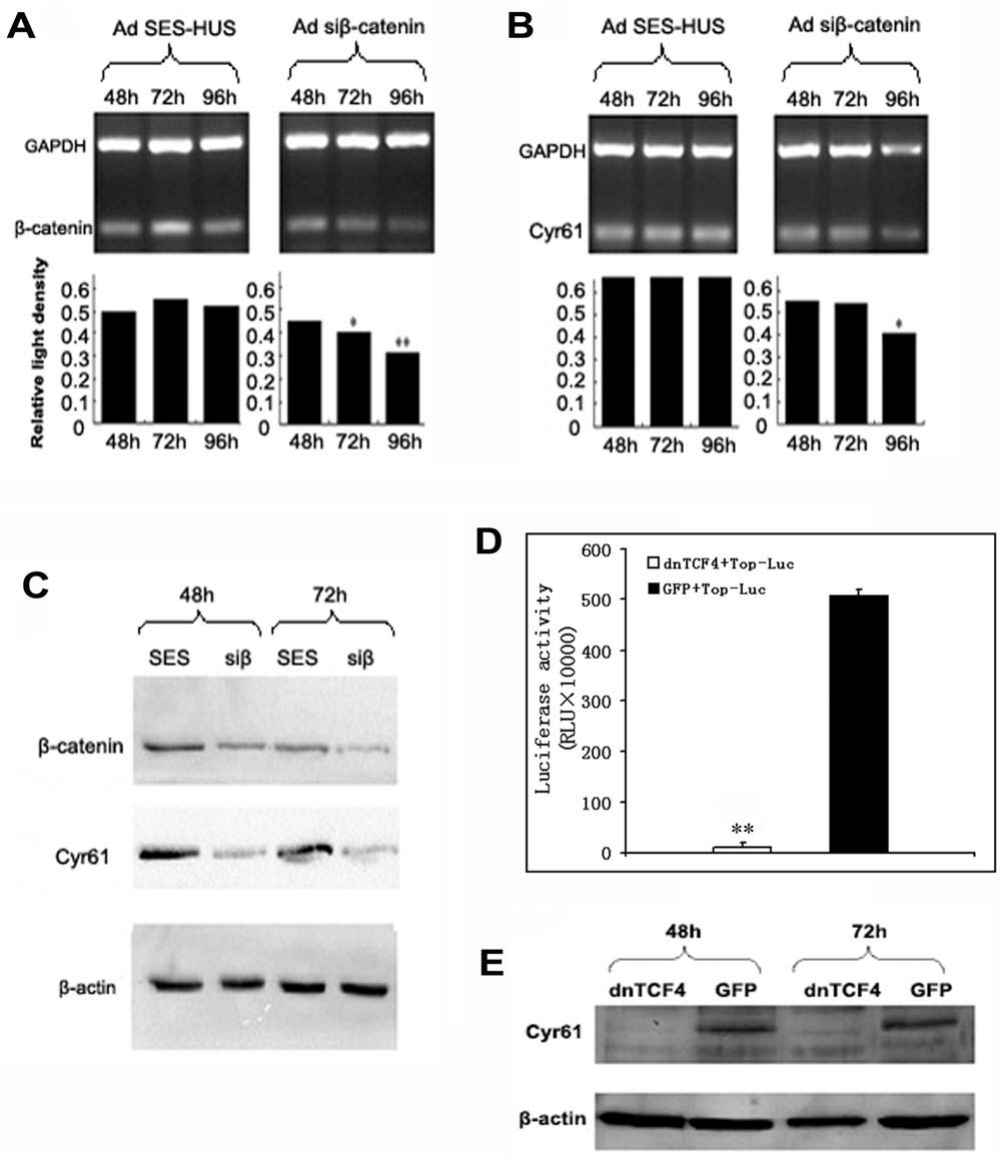
Inhibition of β-catenin down-regulates expression of Cyr61. HepG2 cells were infected with Adsiβ-catenin or AdSES-hus at 24 hrs after plating. Cells were harvested at 48, 72, and 96 hours after continuous incubation; RT-PCR and Western-blot were performed to assess the mRNA and protein levels of Cyr61 and β-catenin. PCR products for β-catenin and GAPDH (**A**) or Cyr61 and GAPDH (**B**) from AdSES-hus and Adsiβ-catenin infected cells at the indicated time points were resolved on agarose gel. Relative quantity of β-catenin or Cyr61 mRNA was calculated based on relative light intensity (β-catenin OD/GAPDH OD or Cyr61 OD/GAPDH OD). *, *P<0.05*, **, *P<0.01*. (**C**), Western blot analysis of β-catenin and Cyr61 in AdSES-hus and Adsiβ-catenin infected cells at the indicated time points. β-actin is used as an internal control. (**D**), Effect of dnTCF4 expression on β-catenin/TCF4 complex activity in HepG2 cells. HepG2 cells were infected with AdTOP-Luc, luciferase activities were measured following AddnTCF4 or AdGFP infection at 30 hours, as described in the Materials and Methods. **, *P<0.01*. (**E**), Expression of Cyr61 protein in response to the change of β-catenin/TCF4 complex activity. HepG2 cells were infected with AddnTCF4 or AdGFP at 24 hrs after plating. Cells were havested at 48 and 72 hours after continuous incubation, and Western-blot was performed to assess the levels of Cyr61 protein. β-actin is used as an internal control.

To further demonstrate that Cyr61 expression is directly related to the levels of β-catenin, we studied the effect of reduced expression of β-catenin on Cyr61 expression. HepG2 cells were infected with adenovirus expressing siRNA for β-catenin (Adsiβ-catenin) or non-specific siRNA (AdSES-hus), expression levels of β-catenin as well as Cyr61 were assessed by RT-PCR and western blot. We found that while both the mRNA and protein levels of β-catenin were reduced by introducing β-catenin siRNA via adenovirus, they were not affected by the non-specific siRNA (Figure 3A, 3C). In cells where the β-catenin was knocked down by siRNA, the mRNA and protein levels of Cyr61 were also decreased, which was not seen in cells with intact β-catenin level(Figure 3B, 3C). These data suggests that β-catenin may be directly responsible for regulating Cyr61 expression.

### β-catenin directly regulates the expression of Cyr61

We then studied whether β-catenin is directly responsible for transcriptional activation of Cry61. β-catenin activates transcription of its target genes through forming a protein complex with TCF, which binds to the promoter region of the target genes. We utilized a dominant negative form of TCF4(dnTCF4) that fails to interact with β-catenin and results in decreased transactivation activity for β-catenin/TCF4 complex and reduced target gene expression. HepG2 cells were co-infected with Luciferase reporter construct AdTOP-Luc and adenovirus expressing dnTCF4 (AddnTCF4) or control virus expressing GFP (AdGFP). A significant down-regulation in TOP-Luc reporter activity was apparent after cells were infected with AddnTCF4, but not the control AdGFP, indicating that the transactivation activity of β-catenin is inhibited in the presence of dnTCF4 (Figure 3D).

We assessed the expression levels of Cyr61 in these cells by western blot. In cells infected with AddnTCF4, where the transactivation activity of β-catenin was inhibited, the Cyr61 protein level was also reduced. This was not seen in cells infected with control AdGFP where the β-catenin activity was not affected (Figure 3E). These findings demonstrated that β-catenin/TCF4 regulates the expression of Cyr61.

**Figure 4:**
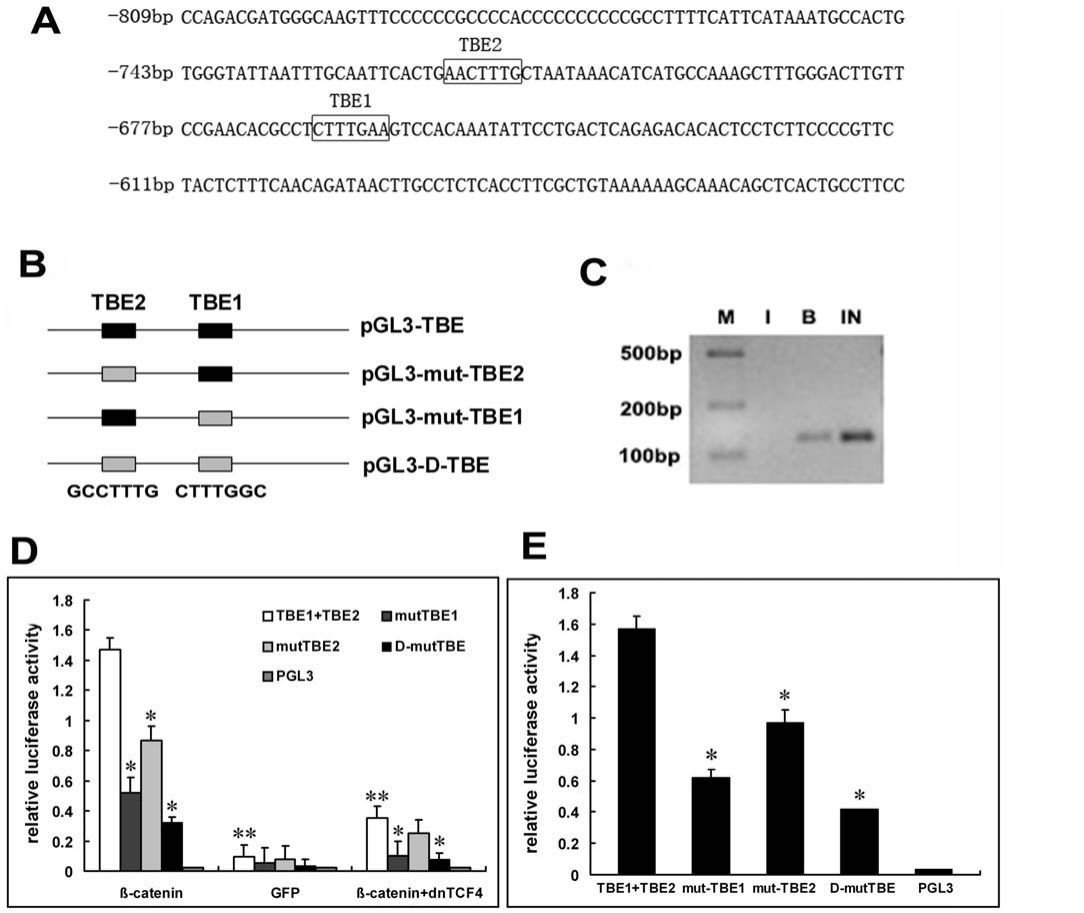
The transcription of human Cyr61 is activated by β-catenin/TCF4 complex. (**A**), Diagram of two putative TBEs in the Cyr61 promoter. TBE1 (CTTTGAA) is located at −660 bp upstream of the transcription start site, whereas TBE2 (AACTTTG) was located at −710 bp upstream of the transcription start site. (**B**), Schematic representation of luciferase reporter constructs from the Cyr61 promoter containing TBEs, TBE mutation constructs (mut-TBE1, mut-TBE2, Double-mutTBE). (**C**), Binding of β-catenin/TCF4 complex to the promoter of Cyr61 in vivo. HepG2 cells were infected with Adβ-catenin for 36 hrs followed standard ChIP procedure using anti-β-catenin antibody or control IgG as described in Material and Methods. The chromatin DNA fragments were used for PCR amplifications with a pair of primers specific for the Cyr61 promoter containing the two TBEs. lane I, IgG; lane B, Anti-β-catenin; In, Input. (**D**), Effect of β-catenin on Cyr61 promoter activity in 293 cells. Cells were co-transfected with luciferase reporter construct and Adβ-catenin, AdGFP, or AddnTCF4, luciferase activities were measured after 30 hours' transfection. Renilla reniformis luciferase reporter (pRL) was used as an internal control. Results were shown as an average of three independent experiments. *, P<0.05, **, P<0.01, compared with pGL3-TBE stimulated with β-catenin. (**E**), Effect of β-catenin on Cyr61 promoter activity in HepG2 cells. Cells were transfected with luciferase reporter construct (pGL3-TBE, pGL3-mutTBE1, pGL3-mutTBE2, pGL3-D-mutTBE), luciferase activities were measured after 30 hours' transfection. Renilla reniformis luciferase reporter (pRL) was used as an internal control. Results were shown as an average of three independent experiments. *, P<0.05 compared with pGL3-TBE.

It has been shown that a consensus TCF4 binding element (TBE) is presented in the target genes of β-catenin/TCF4 complex [29], [30], [31]. We identified two putative TBEs in the human Cyr61 promoter region. TBE1 (CTTTGAA) and TBE2 (AACTTTG) are located at −660 bp and −710 bp upstream of the transcription start site respectively (Figure 4A).

ChIP assay was employed to determine whether β-catenin/TCF4 directly bind to the promoter region of Cyr61 in HepG2 cells. Agarose gel analysis of PCR products showed that anti-β-catenin antibody effectively immunoprecipitated the Cyr61 promoter containing the two TBEs (Figure 4C, lane B). The interaction between β-catenin and the Cyr61 promoter sequence was specific, as only β-catenin antibody but not control IgG was able to pull down the Cyr61 promoter DNA fragments (Figure 4C, lane B,I).

To determine if over-expression of β-catenin affected Cyr61 promoter activity directly, the fragment extending from −750 bp to −500 bp upstream of the transcriptional start site of human Cyr61 promoter was cloned into pGL3 to generate a luciferase reporter construct (pGL3-TBE). As shown in Figure 4D, over-expression of β-catenin significantly activates Cyr61 promoter as indicated by the increased luciferase activity compared with the control in 293 cells. DnTCF4 was able to abrogate this activation since the luciferase activity was greatly reduced in cells when Ad-dnTCF4 was cotransfected with Adβ-catenin.

Furthermore, to determine the role of TBE motifs in regulating Cyr61 gene transcription, site-directed mutation of one or double TBE sites was generated in the context of pGL3 luciferase reporter plasmid. Cyr61 promoter activity, which was increased by β-catenin in 293 cells, was abrogated when one or both of the TBE sites were mutated (Figure 4D). Cyr61 promoter activity was decreased when either of the TBE site was mutated in HepG2 cell lines (Figure 4E). And the promoter activity was markedly reduced if both of the TBE1 and TBE2 sites were mutated (Figure 4E). These data demonstrated that β-catenin activates Cyr61 promoter directly.

### Cyr61 promotes the growth of HCC xenografts in SCID mice

**Figure 5:**
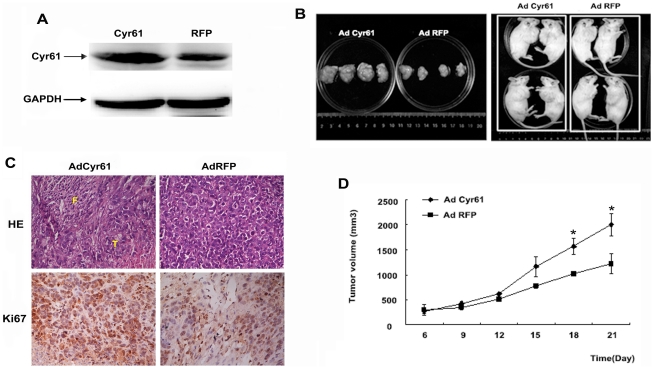
Cyr61 promotes growth of HCC xenograft. HCC xenograft models were established by subcutaneous injection of HepG2 cells infected with AdCyr61 or AdRFP for 36 hrs prior to injection. Two groups of SCID mice (n = 4/group) were injected with 5.0×10^6^ cells per mouse. (**A**), Cyr61 levels in HepG2 cells infected with AdCyr61 and AdRFP. (**B**), Animals and their tumor tissues at 21 days after injection. (**C**), H&E staining of tumor tissues showed fibrous connective tissues hyperplasia, inflammatory cell infiltrating and tumor cells with multinuclear in xenograft tumor samples (400×), F: fibrillar connective tissue, T: tumor. Immunohistochemical detection of Ki67 in xenograft tumor samples (400×). (**D**), Tumor sizes were measured at a 3-day interval starting from day 6 after injection. The graph shows the tumor volume for each treatment group. *, *P<0.05*.

Previously, we found that over-expressing the exogenous Cyr61 in HepG2 cells by recombinant adenovirus vector leads to increased proliferation and migration ability in HepG2 cells [27]. Our results suggested a role of Cyr61 in promoting HCC tumor genesis. In this study, we further investigated the role of Cyr61 on tumor growth *in vivo* with an animal model. HepG2 cells were infected with AdRFP or AdCyr61 at the same infection ratio for 36 hrs before subcutaneous implantation. Tumor sizes were measured every 3 days after implantation. As seen in Figure 5B and 5D, the tumor of HepG2 cells infected with AdCyr61 grew significantly faster *in vivo* than that of the HepG2 cells infected with AdRFP. Over-expression of Cyr61 increased the growth rate by 22% compared with the RFP control group (*P* = 0.02). Specifically, the doubling times for the tumor mass of Cyr61 over-expressed cells and RFP control cells were 2.53±0.16 days and 3.47±0.27 days respectively in the HepG2 xenografts in SCID mice.

H&E staining of the xenograft tumor tissue revealed that there were hyperplasia in fibrous connective tissue, infiltration of inflammatory cells, and multinuclear tumor cells (Figure 5C). To explore whether Cyr61 promotes HCC xenografts by inducing cell proliferation, we assessed the expression of Ki-67, a nuclear protein that is necessary for tumor cell proliferation, in HCC xenograft tissue by immunohistochemical staining. The positive staining of Ki-67 was stronger in the xenograft tissues from the experimental group than those from the control group (Figure 5C).

## Discussion

Given the importance of Wnt/β-catenin pathway in normal embryonic and adult liver development, it is not surprising to see that its activity is perturbed in HCC. About 50%–70% of HCC are found to have an increased level of β-catenin in the cytoplasm or nucleus, which is believed to provide growth advantage for tumor cells. Many of the target genes of Wnt/β-catenin signaling pathway are involved in promoting cell proliferation.

In the present study, we showed that Cyr61 is over-expressed in HCC, and that it is one of the target genes for Wnt/β-catenin pathway. We also demonstrated that increased expression of Cyr61 promoted the growth of HepG2 cell xenografts in SCID mice.

The roles of the Cyr61 in cancer development are complex. A higher level of Cyr61 is found in breast cancer and it is shown to induce estrogen-independence and to promote invasiveness of breast cancer [32]. Cyr61 is shown to have elevated mRNA and protein levels in pancreatic cancer [33]. However, in endometrial tumor, Cyr61 level is decreased compared to normal endometrium [18]. It is also reported that a high Cyr61 level is associated with a lower risk of recurrence of prostate cancer after surgery [15]. The involvement of Cyr61 in tumorigenesis has been mentioned in other investigations. Its exact role is not conclusive. Cyr61 is critical for pancreatic carcinogenesis through inducing EMT and stemness [33]. Cyr61 promotes colony formation and cell growth in esophageal squamous cell carcinoma [34]. However, it has also been reported that Cyr61 suppresses the growth of non-small-cell lung cancer cells [16].

In our study, it is worth noticing that Cyr61 is expressed markedly higher in cancer-adjacent hepatic cirrhosis tissue, which was recognized as precancerous lesions, than in the tumor tissue itself. These results suggest that the abnormal expression of Cyr61 may be closely related to the development of HCC and hepatic cirrhosis, and Cyr61 may be involved in the progression of hepatic cirrhosis to HCC. It is also noted that the expression of Cyr61 is reduced in poorly differentiated HCC. Similar findings are seen in colorectal cancer [35] and gastric cancer [36], in which Cyr61 is over-expressed, while its expression is reduced in more advanced cancer. This data suggested that Cyr61 might be a helpful early diagnosis marker for HCC and one of the indicators for the transformation of liver cirrhosis to HCC.

The exact role of Cyr61 in human cancers remains largely undefined. Both inhibitory and promoting effects of Cyr61 on cancers have been reported. We demonstrated in this study that Cyr61 protein expression was increased in HCC, which is consistent with our previous work and that of the others [22], [28]. However, some other studies found that Cyr61 was down-regulated in HCC tumor tissues by quantitative real-time PCR [19] or cDNA array [37] analysis. There were also studies showing that no significant expression of Cyr61 was detected in HCC compared to the normal tissue [21], [38]. The reasons for the discrepancy can be many folds. Difference in recognition of the boundaries between tumor tissue and tumor-adjacent normal liver tissue may produce different results among the laboratories. Regardless that we demonstrated in our studies that HCC of hepatic cirrhosis or non-cirrhosis origin and the stages of cancer influence Cyr61 expression. These variations, however, are not considered in the other reported studies. Experimentally, we studied both the mRNA and protein levels of Cyr61 in HCC, HCC adjacent tissue and normal liver tissue.

Feng et al showed that Cyr61 suppressed proliferation of HCC cell line, and negatively regulated cell motility and invasiveness. The author concluded that Cyr61 acts as a tumor suppressor in HCC [19]. Our previously published data had shown that over-expression of exogenous Cyr61 promoted the proliferation and migration of HepG2 cells [27]. In the present study, we have repeated our experiments for the effect of Cyr61 on the growth of HepG2 cells xenografts in SCID mice. Our results indicate that Cyr61 can promote the proliferation of HepG2 cells not only *in vitro* but also *in vivo*. Animal experiments are in general considered as a better assessment for tumor development than the *in vitro* experiments. Overall, we believe that our conclusion is supported by our *in vitro* and *in vivo* findings.

Cyr61 is known to interact with various integrins, including α_γ_β_3_, α_6_β_1_, α_γ_β_5_ and α_II_β_3_, which leads to a variety of biological activities, including cell adhesion, migration, and invasion [39]. Furthermore, several studies showed that Cyr61 can be activated under hypoxia or trauma and is involved in angiogenesis [40] and cancer cell invasion [41]. The complex function of Cyr61 makes it difficult to simply define it as an oncogene or a tumor suppressor gene in the development of hepatocellular carcinoma. It likely has a variety of functions in the development of HCC at different stages and may work with a variety of factors.

In summary, our study indicates that Cyr61 is a target gene of β-catenin signaling in HCC. Over-expression of Cyr61 is induced by abnormal expression of β-catenin and promotes the growth and migration of HCC cell line *in vitro* and the progression of HCC xenografts in SCID mice. Based on our results, it is reasonable to hypothesize that early interference with Cyr61 signaling pathway may prevent the transition from hepatic dysplasia to HCC. Future studies about the complex role and mechanism of Cyr61 in tumorigenesis and progression of HCC are needed.

## Materials and Methods

### Ethics Statement

The experimental protocols were approved by the Ethical Committee of Third Military Medical University (TMMU). Verbal consent was obtained from all patients and guardians prior to the procedure. Written consent, which contains many personal information, may impact the lives of patients. So, we think that written consent was not necessary. This decision was approved by the Ethical Committee of Third Military Medical University.

All animal protocols were approved by the Ethical Committee of Third Military Medical University (approval number: 2009020015).

### Human tumor samples

All patients samples were collected from the First Affiliated Hospital of TMMU. Complete clinical data and pathological grade were also available for all patients. Among the collected samples, 35 cases (30 male and 5 female) of HCC, with patients aging from 28 to 73 years old (an average of 49.8), were paraffin-embedded tissues. 30 cases (male) of hepatic cirrhosis of paraffin-embedded cancer-adjacent tissues, aging from 39 to 67 years old (an average of 50) were collected from the Department of Pathology. All these specimens were subjected to histological examination and immunohistochemistry studies. 27 cases of surgical resection HCC and HCC adjacent specimens (23 male and 4 female), with age ranging from 26–68 years old (an average of 50), were acquired from the Department of Hepatobiliary Surgery. Five cases of normal liver samples were obtained from deceased liver donors. Liver tissue samples were fixed in 4% formalin for histological examination and immunohistochemistry, and were used to justify for final diagnoses. None of the patients had received preoperative chemotherapy or radiation therapy.

### Cell lines and cell culture

The cell lines HepG2 and HEK293 were obtained from Shanghai Cell Bank (Shanghai). HepG2 cells were cultured in RPMI1640 supplemented with 10% heat-inactivated fetal calf serum, 100 units/ml penicillin and 10 µg/ml streptomycin at 37°C in a humidified 5% carbon dioxide atmosphere. HEK293 cell line was cultured in DMEM supplemented with 10% heat-inactivated fetal calf serum, 100 units/ml penicillin and 10 µg/ml streptomycin at 37°C in a humidified 5% carbon dioxide atmosphere.

### Immunohistochemical staining

Analysis of β-catenin and Cyr61(Abcam) expression was performed on the paraffin section of human liver tissues. The staining procedure was conducted as decribed previously [28]. Slides were evaluated using a standard bright field microscope. Digital images were then acquired using the NIS-Element Imaging Analysis System (Nikon, Japan). Positive staining was defined as more than 10% of cells containing brown color. The degree of the staining was digitalized automatically using NIS-Element Imaging Analysis System once the positive and negative points were defined. The mean density of random five microscopic fields was calculated which reflects the relative expression level of β-catenin or Cyr61. Results were shown as (mean±S.D).

### Transient infections

HepG2 cells were seeded in six-well plates. At 80% confluence, cells were infected with adenovirus expressing various genes including Adβ-catenin or AdGFP, Adsiβ-catenin or AdSES-hus (negative control siRNA), AddnTCF4 or AdGFP, AdCyr61 or AdRFP, respectively. After infection, cells were harvested at 48 h, 72 h and 96 h for subsequent analysis.

### Plasmid constructs and reporter assays

To determine if over-expression of β-catenin affected Cyr61 promoter activity directly, we construct a luciferase reporter pGL3-TBE. The fragment extending from −750 bp to −600 bp upstream of the transcriptional start site of human Cyr61 promoter, which contains two TBEs, was generated by PCR amplification with the following primers: sense: 5′-CGGGGTACCATGCCACTGTGGGTATTAATTTG-3′, anti-sense: 5′-TCCCCCGGGTGA AAGAGTAGAACGGGGAAGA-3′. The PCR product was cloned into pGL3 using *KpnI* and *SmaI*. Point mutations in the putative TCF-binding site(TBE) were generated from the pGL3-TBE luciferase reporter plasmid according to the manufacturer's recommendations (Stratagene, QuikChange mutagenesis kit). 293 cells were seeded in twenty-four-well plates and cultured up to 80% confluence, at which point they were infected with Adβ-catenin, AdGFP, or AddnTCF4, and at the same time, transfected with a luciferase reporter construct (pGL3-TBE, pGL3-mutTBE1, pGL3-mutTBE2, pGL3-D-mutTBE) using Lipofect2000. HepG2 cells were seeded in twenty-four-well plates and cultured up to 80% confluence, at which point they were transfected with a luciferase reporter construct (pGL3-TBE, pGL3-mutTBE1, pGL3-mutTBE2, pGL3-D-mutTBE) using Lipofect2000. Renilla luciferase reporter was used as internal control. Thirty hours after infection, luciferase assay was performed using the Dual Luciferase Assay System Kit (Promega). All experiments were performed in triplicate. Results are shown in means ± S.D.

### Luciferase Reporter Assay

Luciferase reporter construct TOP-Luc containing three copies of the Tcf/Lef sites upstream of a thymidine kinase (TK) promoter and the firefly luciferase gene(AdTOP-Luc) was used to examined the effect of the dominant negative TCF4 (dnTCF4) on β-catenin/TCF4 transactivation activity. HepG2 cells were seeded in 24-well plates. At 80% confluence, cells were infected with AddnTCF4 or AdGFP. Twenty-four hours after infection, cells were infected with AdTOP-Luc. Thirty hours after infection, luciferase assays were performed, using Luciferase Assay System kit, in accordance with the manufacturer's protocols (Promega). All experiments were performed in triplicate. Results are shown in means ± S.D.

### Reverse Transcription-PCR (RT-PCR)

cDNA was synthesized using oligo (dT_20_) with a Superscript RT-PCR kit (TOYOBO Japan). The generated cDNA was amplified using gene-specific primers: β-catenin 5′-AAAGCGGCTGTTAGTCACTGG-3′ and 5′-GACTTGGGAGGTATCCACATCC-3′; Cyr61 5′-ACTTCATGGTCCCAGTGCTC-3′ and 5′-AAATCCGGGTTTCTTTCACA-3′; GAPDH 5′-CATCATCTTCCAGGAGCG-3′ and 5′-TGACCTTGCCCACAGCCTTG-3′.

### Western blot analysis

Cell lysates were prepared with cell lysis buffer containing a protease inhibitor cocktail (Roche). 50 µg of total protein for each sample was loaded onto 8% SDS-PAGE and transferred to PVDF membrane. The membrane was incubated with antibody against β-catenin or Cyr61 at a dilution of 1∶1000 or 1∶2000 respectively. After the blots were incubated with horseradish peroxidase-conjugated secondary antibody, immune-reactive signals were detected using ECL kit (Millipore America).

### Chromatin immunoprecipitation assay

Chromatin immunoprecipitation (ChIP) assay was carried out as described previously [25]. A pair of primers specific for the human Cyr61 promoter, located from −750 bp to −600 bp relative to the transcription start site of Cyr61, were used for PCR amplification. Primer was 5′-ATGCCACTGTGGGTATTAATTTG-3′ and 5′-TGAATGAAAGAGTAGAACGGGGAAG A-3′.

### Animal models experiments

After infected with AdCyr61 or AdRFP for 36 h, HepG2 cells were subcutaneously injected into the flank of 4-week-old SCID mice (Experimental Animal Center, Third Military Medical University, ChongQing, China), at 5.0×10^6^ cells per mouse. Tumor size of each mouse was measured every three days regularly, and the tumor volume was estimated with the formula *a*×*b* ^2^×0.5, where *a* and *b* represent the maximal and minimal diameters respectively. Mice were euthanized on day 21, and tumor masses were retrieved for histological analysis and immunohistochemical staining of Ki67. Growth curves were plotted using tumor volume for each experimental group at the set points.

### Statistical analysis

Data is expressed as mean ± standard deviation (S.D.). Statistical analysis of the data was performed with the Student paired t test, the 2-tailed Student t test at a statistical level of *P*<0.05 or *P*<0.01. The average absorption of the immunohistochemistry staining for the matched patient samples was compared using the *K* Pearson test at a statistical level of *P*<0.01. SPSS software version 13.0 was used to analyze these data.
